# Supplementary material for: A Lesion-Based Convolutional Neural Network Improves Endoscopic Detection and Depth Prediction of Early Gastric Cancer
Source: J Clin Med. 2019 Aug 26;8(9):1310. doi: 10.3390/jcm8091310 (PMC6781189; doi:10.3390/jcm8091310)
Supplement: Supplementary file 1 [file jcm-08-01310-s001.zip › jcm-558171-supplementary/supplementary full _revision (image remove) .docx]

**Supplementary Materials**

***Lesion-based training***

To detect EGCs and predict their depth, we used the transfer learning method with a VGG-16 network, which is a well-known model comprising 16 convolutional layers and a rectified linear unit. The last three convolutional layers are fully connected to their output. We adapted the last layer of VGG-16 to a two-dimensional, fully connected layer to classify the input endoscopy image into two classes (EGC *vs*. non-EGC and T1a-EGC *vs*. T1b-EGC). A two-way softmax layer was connected to the last fully connected layer to set the output classification probability to [0, 1]. First, to initialize the weights of the network, VGG-16 was trained on the ImageNet classification dataset. Subsequently, all layer weights were fine-tuned by learning our dataset.

Most existing classification models were trained to minimize only one loss of the classification results, such as the cross-entropy loss ^1-2^. However, there are limitations in obtaining high classification performances by optimizing an objective function with the classification loss. To precisely detect EGCs and predict their depth, we proposed a loss function that jointly measures the classification loss of probability and localization loss of the activated regions. The trained model using Grad-CAM loss was defined as the lesion-based network in this study (lesion-based VGG-16). We used the cross-entropy loss $\mathcal{L}_{C}$ for the classification loss and Grad-CAM loss $\mathcal{L}_{G}$ based on Grad-CAM, which visualizes the activated regions of the convolutional layer, for the localization loss ^3^. Cross-entropy loss has been used to measure the performance of a classification model that outputs probability values for each target class between 0 and 1. If the predicted probability diverges from the actual class of an input image, cross entropy loss will be larger according to the following equation:

$$\mathcal{L}_{C}=-\sum_{c=1}^{M} y_{c}\left( x \right)\log p\left( c|x, \mathcal{W} \right),$$

where $y_{c}(x)$ and $p\left( c|x, \mathcal{W} \right)$ are the actual label and predicted probability defined by model weights $\mathcal{W}$ of input EGC image $x$ for class $c$. $M$ denotes the number of target classes (e.g., $M=2$ for EGC vs. non-EGC classification model). Grad-CAM of the last convolutional layer was used to measure Grad-CAM loss. The low activation value indicated does not mean complete exclusion from the features, as the activation values are relative in an image. Activated regions with high value were more often used as visual features in the following fully connected layers. Grad-CAM loss was measured as the dice coefficient loss between the gold-standard EGC binary mask and Grad-CAM output from the last convolutional layer as follows:

$$\mathcal{L}_{G}=-\sum_{c=1}^{M} \frac{2\times\left| P_{c}^{G}(x)\cap M_{c}\left( x \right) \right|}{\left| P_{c}^{G}(x)\cup M_{c}\left( x \right) \right|},$$

where $P_{c}^{G}(x)$ and $M_{c}\left( x \right)$ indicate Grad-CAM consisting of activation values for class $c$ from the last convolutional layer and binary mask of actual EGC regions of the input endoscopy image $x$, respectively ^4^. The final loss $\mathcal{L}$ was computed by a weighted summation of cross-entropy and Grad-CAM losses, as follows:

$$\mathcal{L=}\alpha\mathcal{L}_{C}+\left( 1-\alpha\right)\mathcal{L}_{G}, 0\leq\alpha\leq1.$$

In this study, we empirically set the weight $\alpha$ to 0.5. The network was optimized using the adaptive moment estimation (Adam) optimizer with an initial learning rate of 1e-5. The training batch size was set to 32 ^5^.

For training the model (solid arrows), the network takes a paired input consisting of an RGB image and a binary mask, where EGC regions are filled with a pixel value of 1. The proposed model takes an RGB image with a size of 224 x 224 x 3 as input. Before entering the input image into the network, the mean value (from ImageNet dataset) subtraction was performed on each color channel of the input RGB image to serve as the distribution of data points to the center (i.e., 0) [2].

We applied two types of data augmentation (image augmentation and mask augmentation) to our training dataset. First, image augmentations including random flip, random small rotation, and random elastic deformation were generated 10 times for each image and mask pair. Next, mask augmentations consisting of random kernel erosions, dilations, and random elastic transforms were applied to the binary masks generated by the image augmentation to reflect the diversity of labeling. Although image augmentation was equally performed on the RGB image and corresponding binary mask pair, mask augmentation was only applied to the binary mask image without transformation of the RGB image. As a result, the prepared training image was augmented 100 times.

We implemented the models on an Intel-Core i7-7700K 4.2 GHz processor with 32 GB RAM and a GeForce Titan XP graphic card with 12 GB CUDA memory. In our batch implementation environment, approximately 40 images were processed per second.

***Invasion depth prediction***

For training, the model requires a binary mask corresponding to the EGC region of an input RGB image to measure Grad-CAM loss; there is no need to feed a binary mask to the trained network for obtaining the classification probability of the test image (inference line of Supplementary Figure 2). Since we set our network to focus on the fine-grained features of EGC regions during training, the network gradually activated cancer regions to the infer class while the input was consecutively propagated. By passing the RGB image to the trained network, we were able to obtain the probability for each class.

***EGC localization***

During the training, the model was trained to properly detect EGCs (or predict their depth) while activating EGC regions by simultaneously optimizing the cross-entropy loss and Grad-CAM loss. By utilizing this characteristic, we segmented the connected components of pixels with an activation value of more than 0.5 as a cancer region localization result. To show the effectiveness of localization of activated regions on the last convolutional layer, we computed the ratio of intersection between the actual EGC region and activated region to the actual EGC region, according to changes in the threshold overlap ratios, which ranged from 0.01 to 1.0 with a step size of 0.01. Among the correctly detected EGCs or predicted depth of EGCs, a correct lesion localization was defined as when the intersection regions over the real EGC region of an input RGB image exceeded the threshold overlap ratio. Consequently, a fraction of the number of images in which the lesions were precisely activated to the number of correctly classified images was computed as a metric.

**Supplementary references**

1. Krizhevsky, A.; Sutskever, I.; Hinton, G. E. In *Imagenet classification with deep convolutional neural networks*, Advances in neural information processing systems, 2012; pp 1097-1105.

2. Simonyan, K.; Zisserman, A., Very deep convolutional networks for large-scale image recognition. *arXiv preprint arXiv:1409.1556* **2014**.

3. Selvaraju, R. R.; Cogswell, M.; Das, A.; Vedantam, R.; Parikh, D.; Batra, D. In *Grad-cam: Visual explanations from deep networks via gradient-based localization*, Proceedings of the IEEE International Conference on Computer Vision, 2017; pp 618-626.

4. Milletari, F.; Navab, N.; Ahmadi, S.-A. In *V-net: Fully convolutional neural networks for volumetric medical image segmentation*, 3D Vision (3DV), 2016 Fourth International Conference on, IEEE: 2016; pp 565-571.

5. Kingma, D. P.; Ba, J., Adam: A method for stochastic optimization. *arXiv preprint arXiv:1412.6980* **2014**.

**Supplementary Table 1.** Groups for 5-fold cross validation.

| **Group** | **Training** | **Validation** | **Test** |
| --- | --- | --- | --- |
| 1 | A,B,C | D | E |
| 2 | B,C,D | E | A |
| 3 | C,D,E | A | B |
| 4 | D,E,A | B | C |
| 5 | E,A,B | C | D |

**Supplementary Table 2.** Composition of the five-fold cross-validation dataset.

|  |  | **Normal** | | **Lesion** | | **Mucosal** | | **Submucosal** | |
| --- | --- | --- | --- | --- | --- | --- | --- | --- | --- |
|  |  | **Cases** | **Images** | Cases | Images | Cases | Images | Cases | Images |
| Cross validation-1 | Train | 405 | 7701 | 420 | 1028 | 236 | 534 | 186 | 494 |
|  | Validation | 51 | 330 | 138 | 330 | 68 | 150 | 61 | 150 |
|  | Test | 54 | 330 | 125 | 330 | 60 | 150 | 63 | 150 |
| Cross validation-2 | Train | 408 | 7712 | 420 | 1026 | 232 | 534 | 188 | 492 |
|  | Validation | 53 | 330 | 124 | 330 | 57 | 150 | 63 | 150 |
|  | Test | 63 | 330 | 139 | 330 | 76 | 150 | 61 | 150 |
| Cross validation-3 | Train | 410 | 7642 | 397 | 1029 | 213 | 546 | 184 | 483 |
|  | Validation | 53 | 330 | 139 | 330 | 76 | 150 | 58 | 150 |
|  | Test | 51 | 330 | 147 | 330 | 74 | 150 | 66 | 150 |
| Cross validation-4 | Train | 399 | 7659 | 403 | 1015 | 215 | 541 | 188 | 474 |
|  | Validation | 52 | 330 | 146 | 330 | 78 | 150 | 64 | 150 |
|  | Test | 66 | 330 | 131 | 330 | 73 | 150 | 57 | 150 |
| Cross validation-5 | Train | 384 | 7638 | 412 | 1017 | 221 | 533 | 191 | 484 |
|  | Validation | 59 | 330 | 130 | 330 | 73 | 150 | 54 | 150 |
|  | Test | 58 | 330 | 139 | 330 | 70 | 150 | 63 | 150 |

**Supplementary Table 3.** Diagnostic accuracy of VGG-16 and lesion-based VGG-16.

|  | **VGG-16** | | **Lesion-based VGG-16** | |
| --- | --- | --- | --- | --- |
|  | **Detecting EGC** | Predicting depth | Detecting EGC | Predicting depth |
| AUC | 0.938 | 0.844 | 0.981 | 0.851 |
| Sensitivity (%) | 80.7 | 81.7 | 91.0 | 79.2 |
| Specificity (%) | 92.5 | 75.4 | 97.6 | 77.8 |
| PPV (%) | 91.9 | 78.0 | 97.5 | 79.3 |
| NPV (%) | 82.0 | 79.3 | 91.1 | 77.7 |

*VGG,* Visual geometry group*; EGC*, early gastric cancer; *AUC,* area under the curves; *PPV* positive predictive value; *NPV* negative predictive value.

**Supplementary Figure legends**

**Figure 1.** Examples of the Grad-CAM output extracted from each convolutional layer of the trained lesion-based VGG-16. The green lines on the first column indicate the actual EGC regions. The images from the second column (the first convolutional layer) to the last column (the last convolutional layer) are the activated map extracted from each convolutional layer of the network. A, T1a-EGC. B, T1b-EGC.

**Figure 2.** Overview of the VGG-16-based model. The solid lines indicate the paths for training the model, while the dotted line indicates the path to infer the class.

**Figure 3.** Localization performances of activated regions extracted using the Grad-CAM method. A, EGC detection models. B, EGC depth prediction model.

**Figure 4.** Example of RGB input images (the first row) and their Grad-CAM results extracted from the last convolutional layer of VGG-16 (the second row) and the lesion-based VGG-16 (the third row). The green lines of the input images indicate the actual EGC regions. In the Grad-CAM outputs, the red color is a higher activation value and the blue color is the opposite. A, EGC detection. B, EGC depth prediction.

**Figure 5.** Comparisons of Grad-CAMs extracted from the last convolutional layer when a network was trained using two types of loss functions. The green lines on the input images indicate the actual EGC regions. A, T1a-EGC. B, T1b-EGC.

**Supplementary Figure 1.**

**Supplementary Figure 2.**

**Supplementary Figure 3.**

**Supplementary Figure 4.**

**Supplementary Figure 5.**
