# Supplementary figures and images for: A Lesion-Based Convolutional Neural Network Improves Endoscopic Detection and Depth Prediction of Early Gastric Cancer
Source: J Clin Med. 2019 Aug 26;8(9):1310. doi: 10.3390/jcm8091310 (PMC6781189; doi:10.3390/jcm8091310)

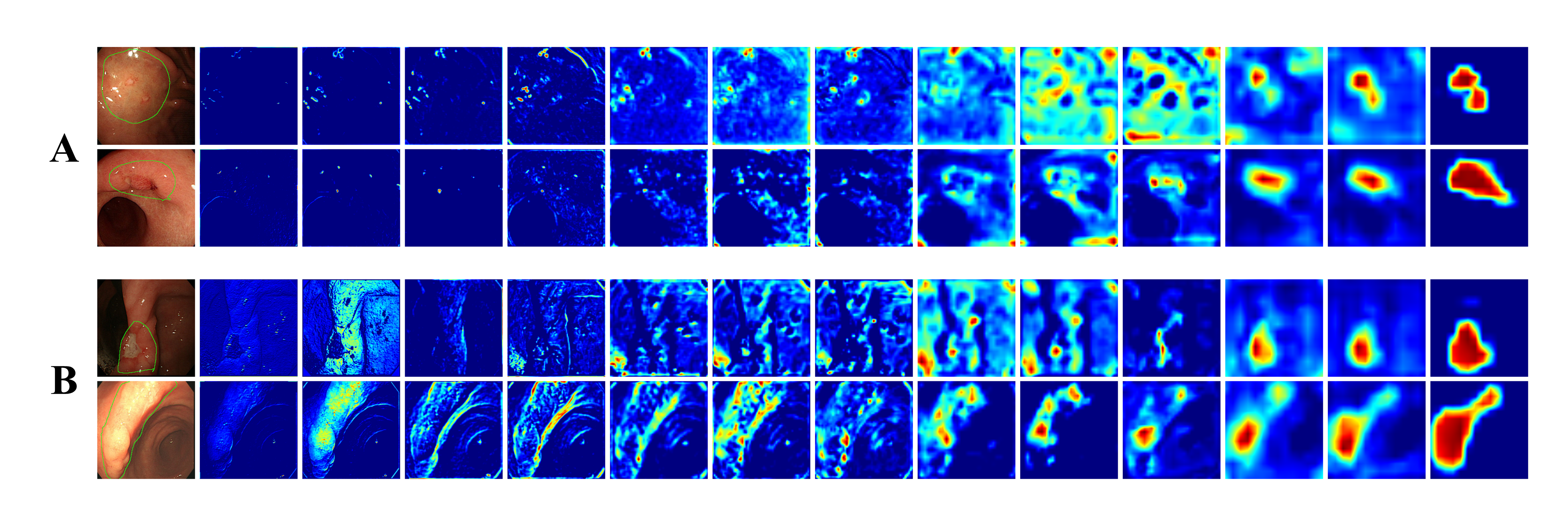

Supplement: Supplementary file 1 [file jcm-08-01310-s001.zip › jcm-558171-supplementary/supplementarry Fig1.tif]

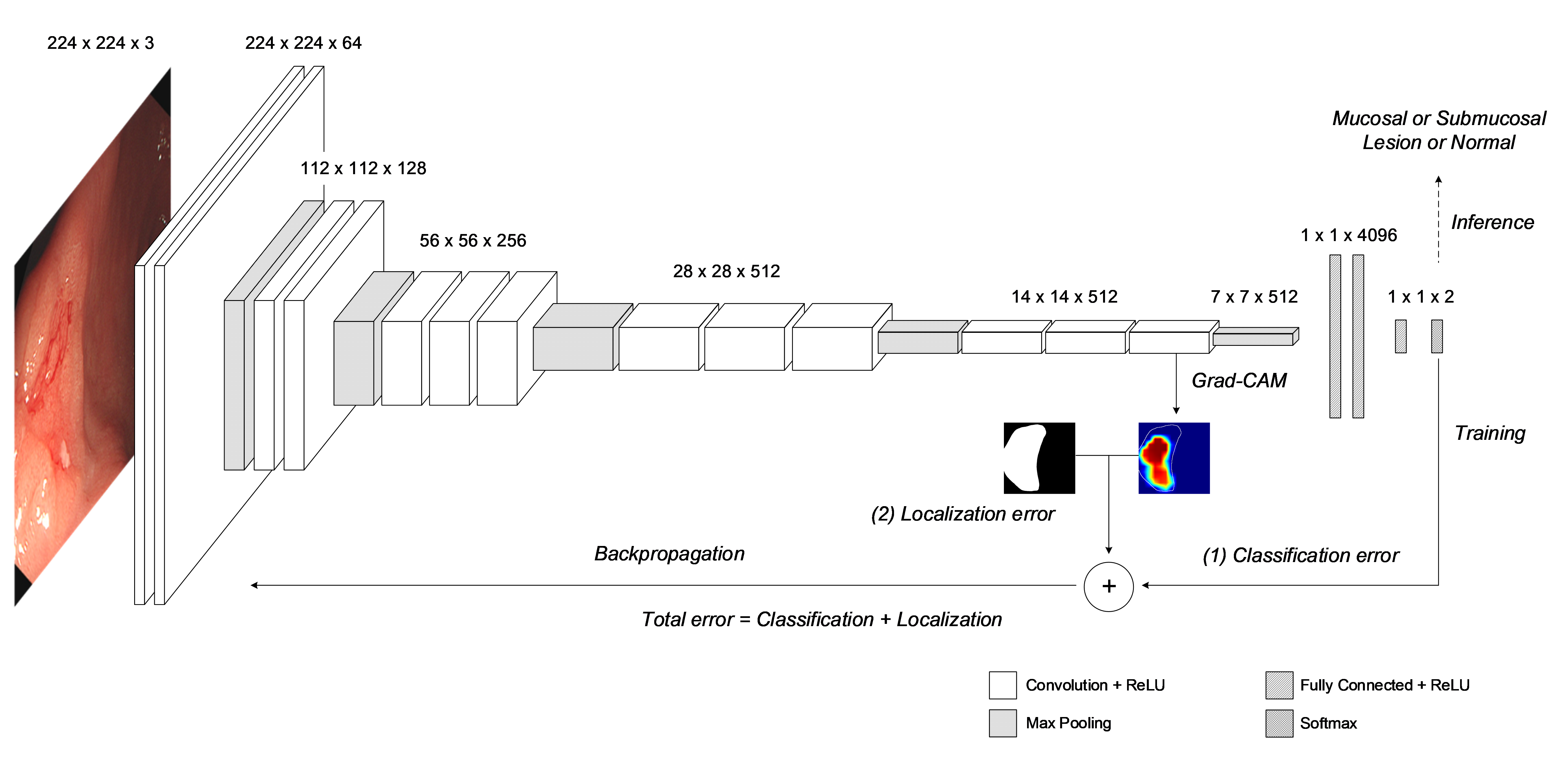

Supplement: Supplementary file 1 [file jcm-08-01310-s001.zip › jcm-558171-supplementary/supplementary Fig2.tif]

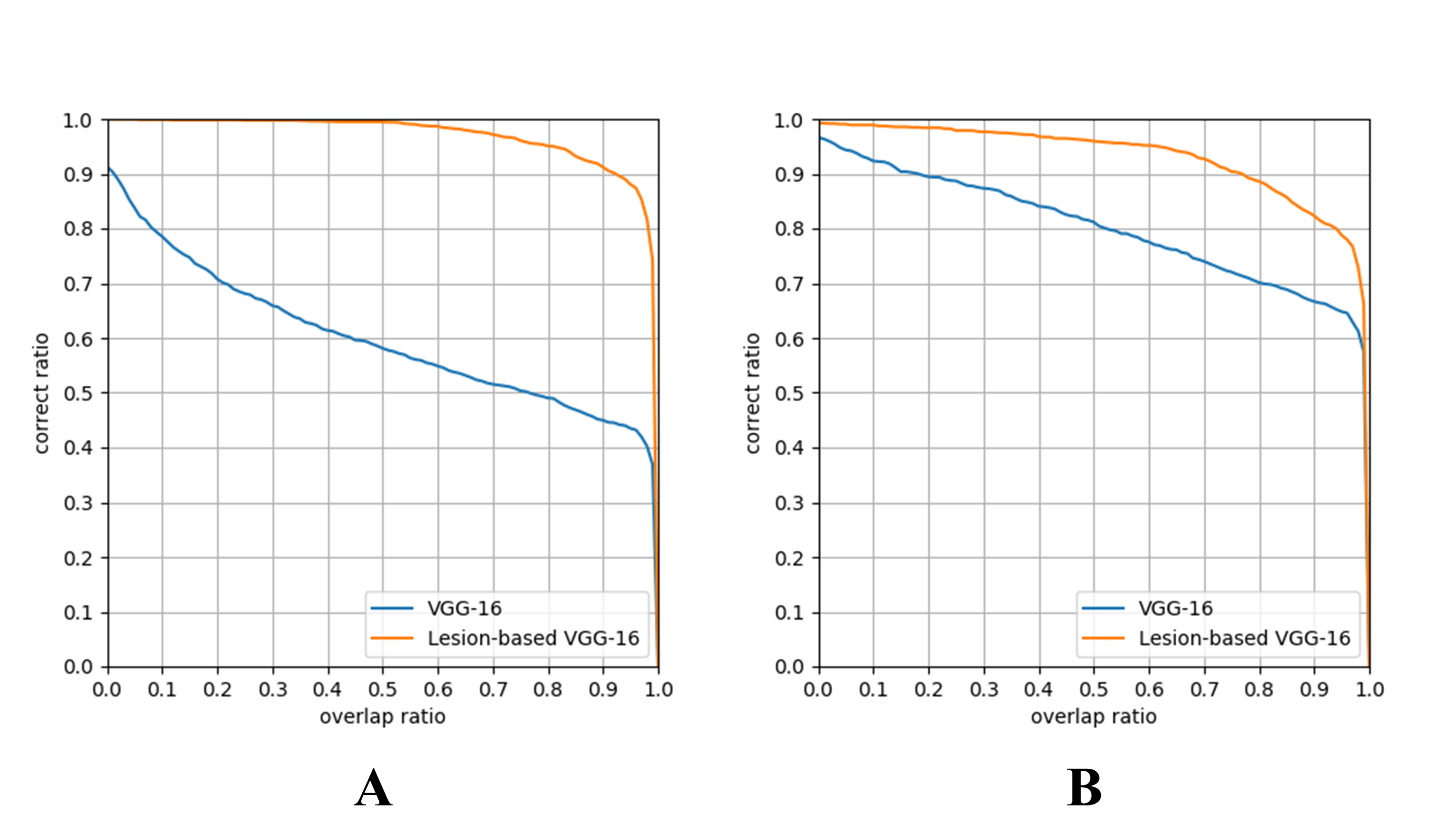

Supplement: Supplementary file 1 [file jcm-08-01310-s001.zip › jcm-558171-supplementary/supplementary Fig3.tif]

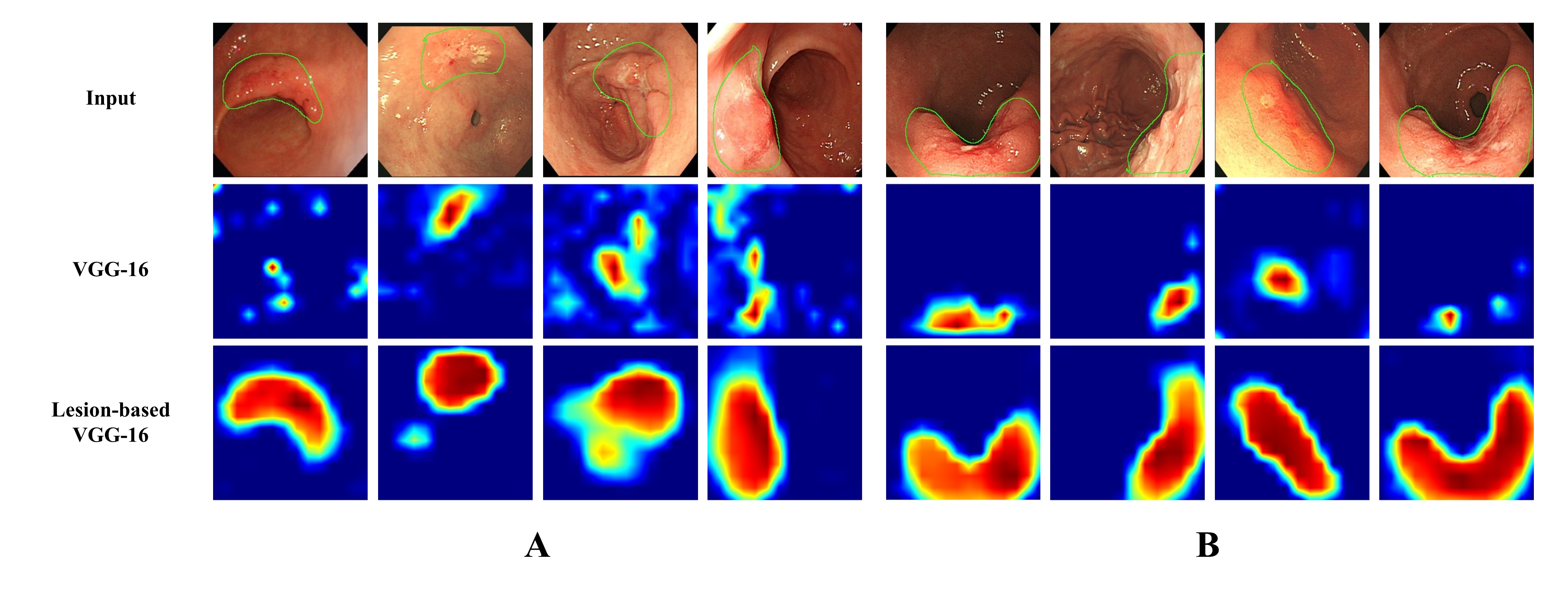

Supplement: Supplementary file 1 [file jcm-08-01310-s001.zip › jcm-558171-supplementary/supplementary Fig4.tif]

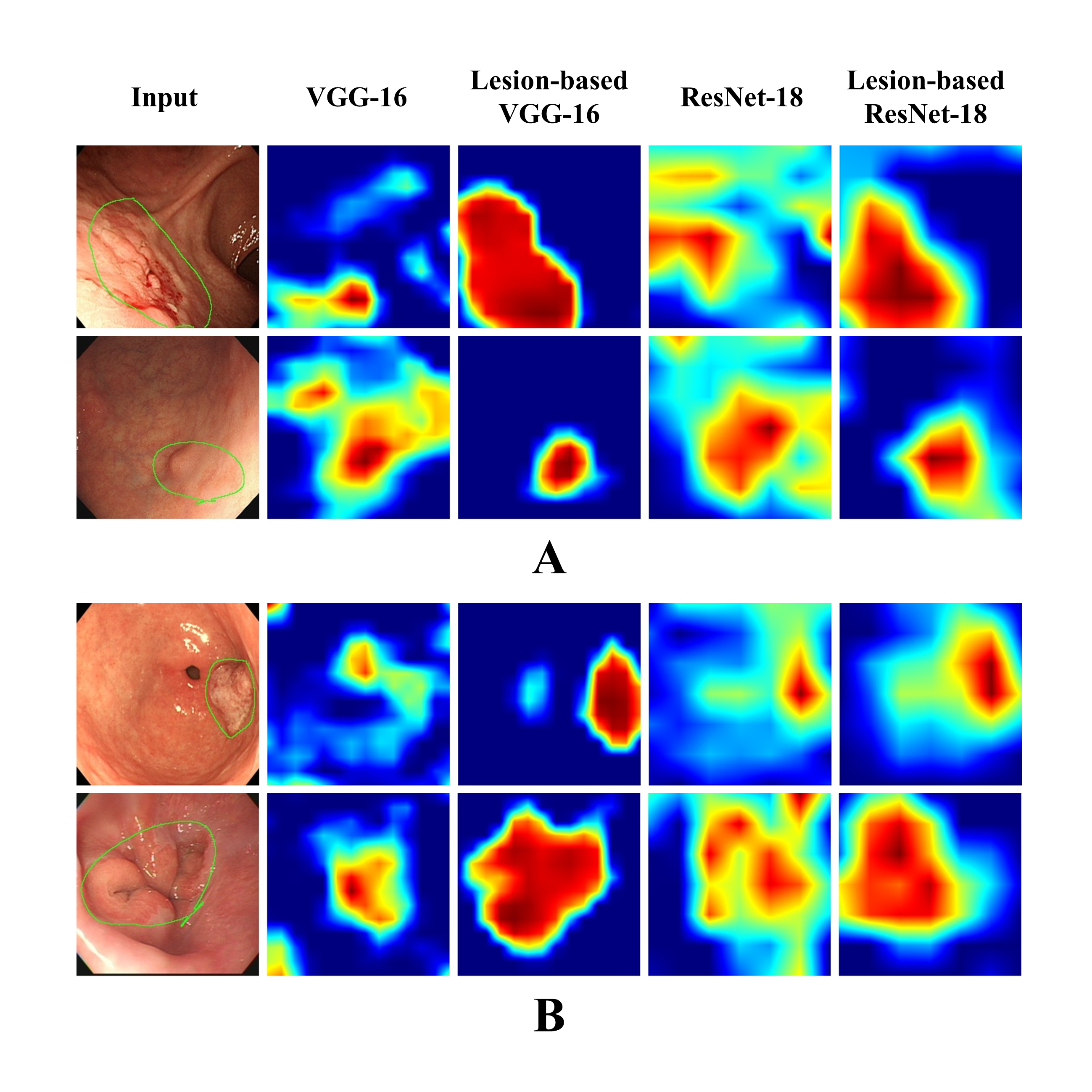

Supplement: Supplementary file 1 [file jcm-08-01310-s001.zip › jcm-558171-supplementary/supplementary Fig5.tif]
